# Segmentation of Portal Vein in Multiphase CTA Image Based on Unsupervised Domain Transfer and Pseudo Label

**DOI:** 10.3390/diagnostics13132250

**Published:** 2023-07-03

**Authors:** Genshen Song, Ziyue Xie, Haoran Wang, Shiman Li, Demin Yao, Shiyao Chen, Yonghong Shi

**Affiliations:** 1 Digital Medical Research Center, School of Basic Medical Sciences, Fudan University, Shanghai 200032, China; gssong20@fudan.edu.cn (G.S.); zyxie22@m.fudan.edu.cn (Z.X.); hrwang20@fudan.edu.cn (H.W.); smli21@m.fudan.edu.cn (S.L.); rh386@sina.com (D.Y.); 2 Shanghai Key Laboratory of Medical Imaging Computing and Computer Assisted Intervention, Shanghai 200032, China; 3 Department of Gastroenterology and Hepatology, Zhongshan Hospital, Fudan University, Shanghai 200032, China; 4 Academy for Engineering & Technology, Fudan University, Shanghai 200433, China

**Keywords:** portal vein segmentation, multiphase CTA image, pseudo label, unsupervised domain transfer

## Abstract

Background: Clinically, physicians diagnose portal vein diseases on abdominal CT angiography (CTA) images scanned in the hepatic arterial phase (H-phase), portal vein phase (P-phase) and equilibrium phase (E-phase) simultaneously. However, existing studies typically segment the portal vein on P-phase images without considering other phase images. Method: We propose a method for segmenting portal veins on multiphase images based on unsupervised domain transfer and pseudo labels by using annotated P-phase images. Firstly, unsupervised domain transfer is performed to make the H-phase and E-phase images of the same patient approach the P-phase image in style, reducing the image differences caused by contrast media. Secondly, the H-phase (or E-phase) image and its style transferred image are input into the segmentation module together with the P-phase image. Under the constraints of pseudo labels, accurate prediction results are obtained. Results: This method was evaluated on the multiphase CTA images of 169 patients. The portal vein segmented from the H-phase and E-phase images achieved DSC values of 0.76 and 0.86 and Jaccard values of 0.61 and 0.76, respectively. Conclusion: The method can automatically segment the portal vein on H-phase and E-phase images when only the portal vein on the P-phase CTA image is annotated, which greatly assists in clinical diagnosis.

## 1. Introduction

The portal vein enters the liver and supplies hepatic blood, accounting for approximately 75% of the total blood supply to the liver [1]. Portal vein thrombosis or cavernous transformation of the portal vein causes obstruction of portal blood flow and reduces hepatic blood supply, which aggravates liver injuries, leads to increased portal vein pressure, and further increases the risk of complications associated with portal hypertension such as ascites, esophageal and gastric varices bleeding, thus affecting the prognosis and quality of life of patients [2,3]. Many studies aim to use machine learning and deep learning methods to segment the liver and related vessels in different modal medical images to promote disease diagnosis, prognosis and surgical planning [4,5,6].

Clinically, multiphase computer tomography angiography (CTA) images are obtained by scanning at different times of peak venous flow. Due to the complementary vessel information on these images presenting a comprehensive status of the collateral circulation, they are routinely used for disease diagnosis and patient prognosis prediction [7]. For example, the diagnosis of portal vein disease often depends on observing the portal vein on the abdominal CTA images of the hepatic artery phase (H-phase), the portal vein phase (P-phase) and the equilibrium phase (E-phase) simultaneously, and intuitively fusing CTA images of different phases, so as to complement vessel information and implement effective treatment [8]. Therefore, it is crucial to accurately segment portal veins on multiphase CTA images.

Due to the different distribution of contrast media between portal veins and surrounding tissues in different phase CTA images, the contrast between the portal vein and the surrounding tissues in the P-phase is the most obvious, which is beneficial for doctors to manually label and construct labeled datasets. Therefore, the existing deep learning methods almost only label the portal veins in P-phase images. Segmenting portal veins on P-phase CTA images is a supervised problem. Supervised methods based on deep learning can generate a best-fit internal representation of the vessel on the image. For example, deep learning has been combined with anatomy for portal vein segmentation in 2D images for liver SBRT planning [9]. As 3D medical images can retain more contextual information, most vessel segmentation algorithms are implemented and improved on the basis of classic 3D segmentation models such as nnUNet and 3D U-Net [10]. For example, Kitrungrotsakul et al. [11] proposed a deep convolutional neural network with multiple pathways called VesselNet for robust segmentation of liver vessels. Yu et al. [12] constructed a novel 3D residual U-Net framework for portal vein segmentation from abdominal CT images. Xu et al. [13] proposed a mean-teacher-assisted confidence learning method for vessel segmentation.

**Figure 1:**
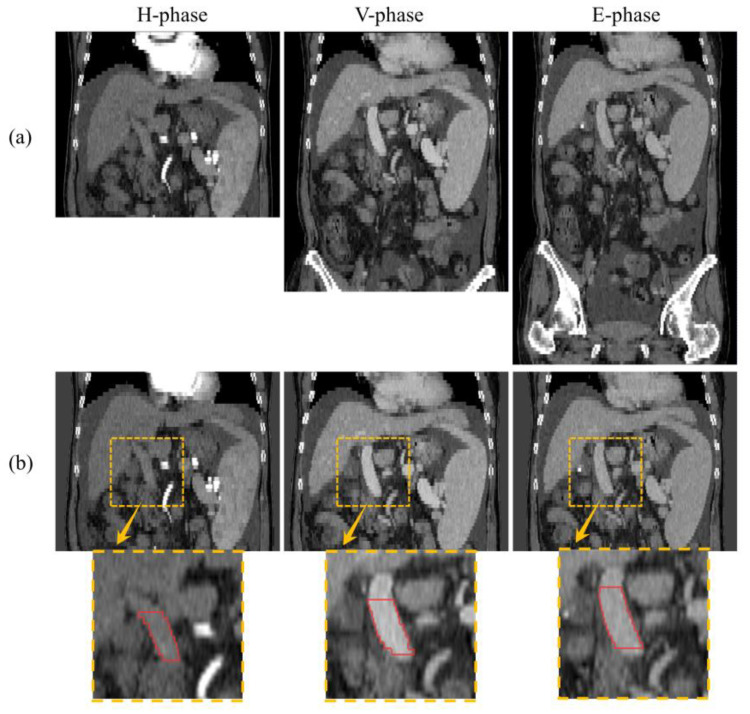
Illustration of the original and aligned CTA images of H-phase, P-phase and E-phase, as well as the magnified portal vein region (orange box) and main portal vein (red curve) in the corresponding images. (**a**) Original image. (**b**) Aligned image.

In order to segment the portal veins from unlabeled H-phase and E-phase images by using the label of P-phase, the existing methods overlap the ground truth of the portal veins in the P-phase images to the corresponding images by simple image alignment for supervised learning. For example, liver segmentation based on EM clustering and GVF level set was performed based on the registration of the multiphase CT dataset [14], and automatic landmark detection and TPS deformation were used for non-rigid registration of multiphase liver CT data [15]. However, these methods cannot accurately reflect the status of the portal vein in different phase images. Specifically, Figure 1 shows the slices of H-phase, P-phase and E-phase CTA images of a subject scanned on the same device, as well as the corresponding portal vein annotation. The orange boxes highlight the portal vein in different phases, while the red curves show the main portal vein. It can be seen that the portal vein is a short and thick main vein [16], which is a smaller target compared to larger organs such as the liver and spleen, making it relatively difficult for physicians to locate the portal vein during observation, especially in H-phase and E-phase images. In addition, the concentration of contrast media in the portal vein varies in the different phases: the contrast between the portal vein and the adjacent tissues is the lowest in H-phase images, the highest in P-phase images and decreases again in E-phase images. To make matters worse, vessels and tissues undergo a certain degree of displacement and distortion between phases. As can be seen, there is an inter-phase shift in the annotation results of the vessels, and the complete portal vein trunk cannot be obtained directly on each phase image by simple alignment.

The above research shows that the existing supervised vascular segmentation deep learning methods mainly focus on changing the network structure, segmenting single-phase images and not exploring cross-phase information. The existing exploration of interphase information utilization, that is, the simple alignment method, ignores the contrast and morphological changes of intertemporal blood vessels. Therefore, we believe that the key to portal vein segmentation in H-phase and E-phase CTA images is to solve the unsupervised domain transfer problem, which is limited by vessel labeling.

It is known that Generative Adversarial Network (GAN) [17] provides a way to learn deep representations without a large amount of annotated training data. And it can generalize the models trained on the source domain (annotated training datasets) to the target domain (test datasets) through transfer learning [18] and visual domain transfer techniques [19]. In particular, the technological breakthrough around GAN, such as CycleGAN [20], can generate high-quality images under unsupervised conditions and is often applied to multimodality image annotation problems. Recently, Xu et al. [13] used noise-labeled data for the challenging task of liver vessel segmentation. Jiang et al. [21] used unpaired CT and MRI images for domain adaptive transformations and guided the student CT networks with the help of an informative teacher MRI network to extract features indicating foreground and background differences. Other studies have explored domain transfer segmentation on CT and MRI images of the heart [22,23]. In general, obtaining the similarity between different images by domain transfer is a feasible annotation method.

In addition, the lack of sufficient public datasets has led to an increasing interest in the study of unsupervised and semi-supervised deep learning [24,25]. Medical images are usually presented in multiple modalities, such as MRI and CT, so unsupervised domain adaptive learning using annotations of one modality to obtain annotations of another modality is a frequently explored topic. For example, Raju et al. [26] proposed the co-heterogeneous and adaptive segmentation (CHASe) method based on liver-enhanced CT images, which requires only a small amount of annotated monophasic data to adapt to any unannotated heterogeneous multiphase data and is highly labor-saving. Qu et al. [27] conducted a similar study on pancreatic-enhanced CT images using two-phase scanning, and their proposed framework can integrate multi-scale and multi-view information for multiphase pancreas segmentation. This study has a strong reference value for the segmentation of portal veins on the multiphase images. Currently, most studies on unsupervised domain adaptation for image segmentation focus on simple tasks such as organs and tumors. Few unsupervised cross-modality studies have been applied to vessel segmentation due to the inconsistent size and a number of vessels observed on CTA images of different phases.

Therefore, for the multiphase CTA dataset of patients with portal-related diseases, based on the annotated P-phase images, this paper proposes a method to segment portal veins on the H-phase and E-phase images based on unsupervised domain transfer and pseudo labels. Firstly, a style transfer network is used to implement modal transformation from H-phase (or E-phase) images to P-phase images, narrowing the style differences between different phases and achieving higher apparent similarity. At the same time, the limitation of insufficient contrast of the portal vein in the original images of the different phases is suppressed on the corresponding ones after style transfer, which is conducive to constructing the pseudo labels. Secondly, the H-phase (or E-phase) images and the style transferred ones that will be input into the 3D U-Net network along with the P-phase images. When training the segmentation model, under the constraint of the ground truths of the portal vein of the P-phase images, the predicted results of the H-phase (or E-phase) images are used to jointly construct pseudo labels with confidence for the corresponding phase. This not only reduces the impact of vessel shift between different phases but also achieves accurate segmentation of portal veins in corresponding phases. Finally, in the case of only one phase image annotation, this method can obtain segmented portal veins on the other two phase images, respectively.

## 2. Materials and Methods

### 2.1. Data Description

The dataset included 169 patients with liver cirrhosis and portal vein disease who visited Zhongshan Hospital, affiliated with Fudan University in Shanghai, China, between January 2016 and May 2020. These patients were scanned for abdominal CTA images in the H-phase, the P-phase and the E-phase. The CTA images were acquired with a delayed scan time of 25 to 30 s for the H-phase, 60 to 70 s for the P-phase and 85 to 90 s for E-phase after contrast media injection. The imaging parameters were as follows: in-plane resolution is between 0.610 mm × 0.610 mm and 0.881 mm × 0.881 mm; slice thickness is 5.0 mm; and the size of the image acquisition matrix is 512 × 512.

Finally, an experienced physician annotated the portal vein trunk on all images.

### 2.2. Image Preprocessing

The preprocessing of CTA images includes removing the CT beds, aligning images of different phases of the same patient, and extracting and normalizing the portal vein blocks.

**Removing the CT beds.** Selecting a threshold of −300 Hounsfield units to binarize the image can remove the CT bed from all images completely and preserve the largest body contour in the images.

**Aligning images of different phases.** Figure 1a shows coronal views of H-phase, P-phase and E-phase CTA images of the same patient. The images of different phases contain different numbers of axial slices, indicating that even for the same patient, it is not possible to determine the location of portal veins in other phases solely based on portal vein annotation in P-phase. According to the body contour extracted by binarization, the slice locations with the largest intersection of the binary images of the three phases were taken so that the images of different phases of the same patient were aligned axially. Figure 1b shows the results of the H-phase, P-phase and E-phase CTA images after the removal of the CT bed and alignment of the images of the three phases.

**Extracting and normalizing the portal vein blocks.** The portal vein occupies a relatively small volume in the whole CTA image and has an irregular shape, especially the weak contrast between the portal vein and the surrounding tissues in H-phase and E-phase images, making it difficult to segment directly in the original image. For this purpose, this study utilizes portal vein annotation in the P-phase image to automatically crop image blocks containing portal veins from the aligned H-phase and E-phase images, with a size of 32 × 128 × 128.

### 2.3. Multiphase Segmentation Network

**Figure 2:**
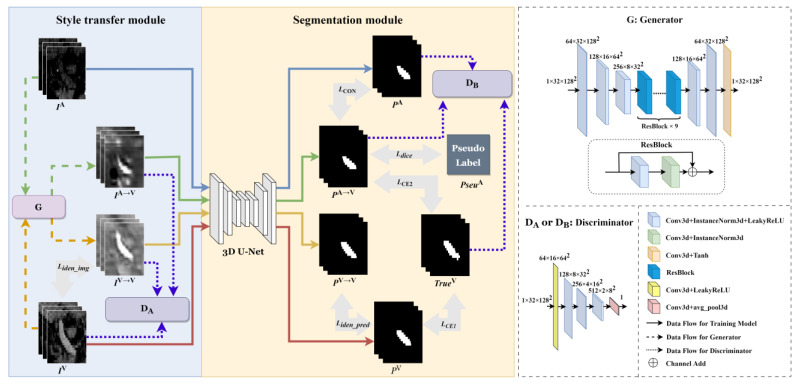
Portal vein segmentation network on multiphase images.

Figure 2 depicts the portal vein segmentation network (called as PVSegNet) on the multiphase images, which consists of two parts: a style transfer module and a segmentation module. The inputs to the network are the H-phase (or E-phase) image, $I^{A}$, and the P-phase image, $I^{V}$. The ground truth of the portal vein in the P-phase image is denoted as ${True}^{V}$. The output of the network is the prediction results of the portal vein on the H-phase (or E-phase) image, $P^{A}$. In the style transfer module, the H-phase (or E-phase) image, $I^{A}$, is transformed to construct the image, $I^{A\to V}$, with a similar style to the P-phase image, $I^{V}$. Similarly, the P-phase image, $I^{V}$, itself can also be transferred to the image, $I^{V\to V}$, with a consistent style. Then, in the segmentation module, these style-transferred images, $I^{A\to V}$ and $I^{V\to V}$, and their original images, $I^{A}$ and $I^{v}$ are trained only under the constraint of the ground truth of the portal vein in the P-phase image, ${True}^{v}$, and the corresponding prediction results are generated to construct pseudo labels for further optimizing the segmentation. Finally, the portal vein on the H-phase (or E-phase) images is segmented automatically and accurately.

#### 2.3.1. Style Transfer Module

Traditional style transfer neural networks are capable of converting information from one representation to another. In order to enable the H-phase (or E-phase) image to have similar characteristics to the P-phase image and segment the portal vein on the corresponding images, inspired by the idea of the domain transfer of the CycleGan network [20], this paper builds the style transfer module composed of a generator and a discriminator, as shown in Figure 2.

First, $I^{A}$ is input into the generator G to generate an image $I^{A\to V}$ with a similar style to $I^{V}$. Since there is a high similarity of organs and tissues between the two-phase images, in order to ensure that the semantic characteristics similar to $I^{V}$ in $I^{A}$ are not changed by the generator and can be preserved, $I^{V}$ also goes through the generator to get $I^{V\to V}$. The image block input to the generator is represented by a tensor with size 1 × 32 × 128 × 128 (representing channel × depth × height × width). These images are sequentially processed through three encoding convolution layers, nine residual structures (ResBlock) and two decoding convolution layers, and the output is the result of style transfer transformation with the same size as the input image. Here, the convolutional kernels of the encoding and decoding layers are connected with InstanceNorm3d and LeakyReLU to form the basic structural module. The size of the convolution kernel is 3 × 3 × 3, and the step sizes of the convolution kernels are all 2.

Secondly, whether the style transfer result $I^{A\to V}$ is similar to $I^{V}$ in terms of style characteristics will be determined by the discriminator D_A_ shown in Figure 2. At the same time, the discriminator also evaluates the transferred image, $I^{V\to V}$, to enhance the ability of the generator to preserve the transformation P-phase image style characteristics. The image block input to the discriminator is represented by a tensor with a size of 1 × 32 × 128 × 128. And these images are sequentially processed through five layers of convolution, and finally, the value 1 or 0 is output by the average pooling (avg_pool3d), which indicates whether the style transfer result is similar to $I^{V}$. The step size of the convolutional kernel in the discriminator network is 2.

It should be noted that in Figure 2, $I^{V}$ is an annotated P-phase image, and $I^{A}$ may be an H-phase or E-phase image to be segmented. This study separately trains the style transfer module on the H-phase or E-phase images and its subsequent segmentation module on the corresponding phase under the guidance of the P-phase images.

#### 2.3.2. Segmentation Module

When the H-phase (or E-phase) images undergo the style transfer transformation and have a similar style as the P-phase image, the segmentation module can segment both the P-phase image $I^{V}$ and its style-transferred image $I^{V\to V}$, as well as the H-phase (or E-phase) image $I^{A}$ and its corresponding style-transferred image $I^{A\to V}$, and output the corresponding prediction results, respectively. The segmentation module consists of a 3D U-Net [10] segmentation network and a discriminator, D_B_, as shown in Figure 2.

**Figure 3:**
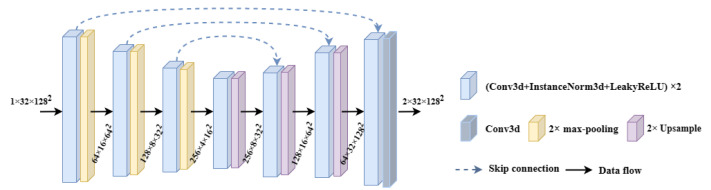
3D U-Net segmentation module.

Figure 3 shows the 3D U-Net network. The image represented by a tensor with size 1 × 32 × 128 × 128 sequentially undergoes four downsamplings (max-pooling) operations with a step size of 2, and four upsampling (ConvTranspose) operations with a step size of 2. And each level’s upsampling layer is directly concatenated with the same level’s downsampling layer through skip connections. The final output is the binary segmentation result of the portal vein on the image. Before each downsampling and upsampling operation, the input image or intermediate calculation results are sequentially extracted at pixel level by two basic structures formed by connecting the convolutional layers, InstanceNorm3d and LeakyReLU.

The discriminator D_B_ of the segmentation module is used to judge whether the output of the 3D U-net network is similar to the ground truth ${True}^{V}$ on the P-phase image, which is an image-level judgment and more suitable as a soft constraint of the segmentation module. The discriminator D_B_ judges the authenticity of the prediction results, $P^{A\to V}$ and $P^{A}$, enhancing the constraint of the network on the final prediction result $P^{A}$. Here, the structure of the discriminator D_B_ is the same as that of D_A_, as shown in Figure 2.

#### 2.3.3. Construction of Pseudo Label

**Figure 4:**
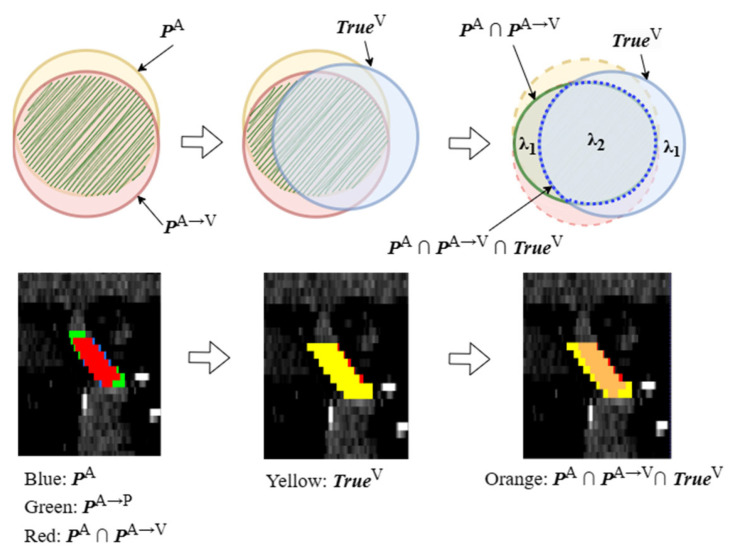
Construction of pseudo label.

During the training process, when using the ground truth ${True}^{V}$ of the P-phase image to guide the segmentation network in training the H-phase (or E-phase) images, it is common to learn incorrect information due to the vessel shift on different phase images. Therefore, this study proposes to use the ground truth of the portal vein in the P-phase image and the prediction results of the segmentation network to jointly construct pseudo labels and guide network training through pseudo labels. Figure 4 shows the process of constructing pseudo labels.

The original H-phase (or E-phase) image $I^{A}$ and its style transferred image $I^{A\to V}$ are input into the 3D U-Net network, and the predicted results $P^{A}$ and $P^{A\to V}$ are obtained. Although they are similar, there are differences between the two. In particular, the intersection area, $P^{A}$∩$P^{A\to V}$, may belong to the possible label area. This intersection area of $P^{A}$∩$P^{A\to V}$ further intersects with the ground truth ${True}^{V}$ of the P-phase image $I^{V}$, and the resulting intersection $P^{A}$∩$P^{A\to V}$∩${True}^{V}$ can be used as the correct label area on image $I^{A}$. The remaining part of ${True}^{V}$ is also considered as a possible label area for image $I^{A\to V}$. Then, set weights $\lambda_{2}-\lambda_{1}$ and $\lambda_{1}$ for the correct and possible label regions separately, and the pseudo labels are calculated as Equation (1):

(1) $${Pseu}^{A}=\lambda_{1}P^{A}\cap P^{A\to V}+\lambda_{1}{True}^{V}+{(\lambda }_{2}{-2\lambda }_{1})\left(P^{A}\cap P^{A\to V}\cap {True}^{V}\right)$$

Here, referring to the overlapping area between the predicted results and the ground truths in Figure 4, $\lambda_{1}$ and $\lambda_{2}$ are set as 0.1 and 1 for the H-phase image, 0.7 and 1 for the E-phase image and 0 for the remaining areas. In addition, Dice loss is set between the pseudo label ${Pseu}^{A}$ and the network prediction result $P^{A\to V}$ for guiding the PVSegNet network to better train and segment $I^{A\to V}$.

#### 2.3.4. Loss Function

The cost function optimized by this method, Loss, is calculated in Equation (2) and consists of five types of the loss function, including Identity loss, $L_{iden\_img}$ and $L_{iden\_pred}$, Consistency loss, $L_{CON}$, Cross entropy loss, $L_{CE1}$ and $L_{CE2}$, Dice Loss, $L_{dice}$, as well as the Discrimination loss, $L_{G\_img}$ and $L_{G\_pred}$.

(2) $$Loss=\omega_{1}L_{iden\_img}+\omega_{2}L_{iden\_pred}+\omega_{3}L_{G\_img}+\omega_{4}L_{G\_pred\;}+\omega_{5}L_{CON}+\omega_{6}L_{CE1}+\omega_{7}L_{dice}+\omega_{8}L_{CE2}$$

where $\omega_{1},\;\omega_{2},\ldots ,\omega_{8}$ represent the weights of the loss functions, respectively, and in this study, $\omega_{1}=\omega_{2}=\omega_{7}=\omega_{8}=5$, $\omega_{3}=\omega_{4}=0.01$, $\omega_{5}=1$, $\omega_{6}=10$.

**Identity loss.** It is generally believed that images should retain their own features after style transfer [20]. This study uses Identity loss to constrain the image $I^{V}$ in order to preserve its own features after transformation by the generator, as shown in Equation (3). $L_{iden\_img}$ measures the difference between the style-transferred image $I^{V\to V}$ and the original image $I^{V}$. In addition, using the same segmentation network to predict the same images should result in the same results. That is to say, the predicted results of images $I^{V}$ and $I^{V\to V}$, $P^{V}$ and $P^{V\to V}$, should also be subject to the constraint of Identity loss, as shown in Equation (4).

(3) $$L_{iden\_img}=\left\{\begin{array}{c} \sum_{i=1}^{H\times W\times D}(I_{i}^{V}-I_{i}^{V\to V}{)}^{2}\;\;\;\;\;\;\;\;\;\;\;if\;|I_{i}^{V}-I_{i}^{V\to V}|<1\\ \sum_{i=1}^{H\times W\times D}|I_{i}^{V}-I_{i}^{V\to V}|-0.5\;\;\;\;\;\;\;\;\;\;\;\;\;\;otherwise \end{array}\right.$$

(4) $${L_{iden\_pred}=\sum }_{c=1}^{C}\left\{\begin{array}{c} \sum_{i=1}^{H\times W\times D}({P^{V}}_{i}^{c}{-P^{V\to V}}_{i}^{c}{)}^{2}\;\;\;\;\;\;\;\;\;if\;|{P^{V}}_{i}^{c}{-P^{V\to V}}_{i}^{c}|<1\\ \sum_{i=1}^{H\times W\times D}|{P^{V}}_{i}^{c}{-P^{V\to V}}_{i}^{c}|-0.5\;\;\;\;\;\;\;\;\;\;\;\;\;\;otherwise \end{array}\right.$$

where C=2 denotes the portal vein and the background. *H*, *W* and *D* represent the height, width and depth of the image, respectively.

**Discrimination loss.** The discriminator network $D_{A}$ in the style transfer module used the binary cross entropy loss function to separate the original P-phase image from the generated P-phase image. When training discriminator $D_{A}$, set the discriminator $D_{A}$ to judge $I^{A\to V}$ as 0, and judge $I^{V}$ and $I^{V\to V}$ as 1.

The discriminator network $D_{B}$ in the segmentation module also optimized the binary cross entropy loss function to discriminate the ground truth ${True}^{V}$ from the predicted results $P^{A\to V}$ or $P^{A}$. When training the discriminator, set the discriminator $D_{B}$ to judge the predicted results $P^{A\to V}$ and $P^{A}$ as 0 and determine that the annotation ${True}^{V}$ of the P-phase image is 1.

In order to ensure that the image $I^{A\to V}$ generated by the generator predicts sufficiently realistic results through the segmentation network so as to deceive the discriminator $D_{A}$ which judges that the generated image $I^{A\to V}$ is 1, and at the same time, makes the discriminator D_B_ determines that the predicted image $P^{A\to V}$ is 1. Therefore, the Discrimination loss functions, i.e., $L_{G\_img}$ and $L_{G\_pred}$, are set respectively in the training process to enhance the effectiveness of the generator and segmentation network, as shown in Equations (5) and (6).

(5) $$L_{G\_img}=-\;log(D(I^{A\to V}))$$

(6) $$L_{G\_pred}=-log(D(P^{A\to V}))$$

where *D*($I^{A\to V}$) and *D*($P^{A\to V}$) represent the binary cross entropy loss results of $I^{A\to V}$ and $P^{A\to V}$ input to the corresponding discriminator, respectively.

**Consistency loss.** In order to form constraints between the original H-phase (or E-phase) image and its style-transferred image and make the segmentation network learn useful knowledge lost due to style transfer, Consistency loss $L_{CON}$ is set between the corresponding prediction results $P^{A}$ and $P^{A\to V}$, as shown in Equation (7).

(7) $$L_{CON}=\sum_{c=1}^{C}\frac{1}{H\times W\times D}\sum_{i=1}^{H\times W\times D}\;({P^{A}}_{i}^{c}{-P^{A\to V}}_{i}^{c}{)}^{2}$$

**Cross entropy loss.** As shown in Equation (8), Cross entropy loss $L_{CE1}$ is set between the segmentation result $P^{V}$ on the P-phase image and the ground truth ${True}^{V}$ to guide the correct convergence of the segmentation network. Multiphase images have similarities, and to some extent, there is information that can be gleaned from one another. Therefore, the Cross entropy loss $L_{CE2}$ is also set to calculate the similarity between the prediction result $P^{A\to V}$ and the ground truth ${True}^{V}$, as shown in Equation (9).

(8) $$L_{CE1}=-\sum_{c=1}^{C}\;\sum_{i=1}^{H\times W\times D}\;{P^{V}}_{i}^{c}log\left(\frac{e^{{{Ture}^{V}}_{i}^{c}}}{\sum_{j=1}^{C}\;e^{j}}\right)$$

(9) $$L_{CE2}=-\sum_{c=1}^{C}\;\sum_{i=1}^{H\times W\times D}{P^{A\to V}}_{i}^{c}log\left(\frac{e^{{{Ture}^{V}}_{i}^{c}}}{\sum_{j=1}^{C}\;e^{j}}\right)$$

**Dice loss.** In addition to the Cross entropy loss, the Dice loss is also set in the segmentation network to reduce the impact caused by the vessel shift between different phases, as shown in Equation (10), which is used to calculate the loss between the prediction result $P^{A\to V}$ and the pseudo label ${Pseu}^{A}$.

(10) $$L_{\mathrm{dice}}=\sum_{c=1}^{C}\;\left(1-\frac{2\sum_{i=1}^{H\times W\times D}\;{P^{A\to V}}_{i}^{c}{{Pseu}^{A}}_{i}^{c}}{\sum_{i=1}^{H\times W\times D}\;{\left({P^{A\to V}}_{i}^{c}\right)}^{2}+\sum_{i=1}^{H\times W\times D}\;{\left({{Pseu}^{A}}_{i}^{c}\right)}^{2}}\right)$$

## 3. Experiments

### 3.1. Experimental Setup and Evaluation Index

The experiments were conducted on Ubuntu 18.04 operating system and PyTorch framework, configured with Intel^®^ Core™ i5-9600K (3.70 GHz × 6 CPUs), 64 GB RAM and one RTX 3090 GPU.

All the experiments are performed using three-fold cross-validation. During the training stage, the H-phase and E-phase training datasets were trained for 50 and 40 epochs to obtain the model with the best performance for testing on the respective test dataset, respectively. The training was performed using the Adam optimizer with an initial learning rate of 0.001.

The evaluation metrics of the segmentation results are standard metrics based on the pixel-level confusion matrix calculation, including the dice similarity coefficient (DSC) [28] and the Jaccard coefficient (Jaccard), as shown in Equations (11) and (12), respectively.

(11) $$DSC=\frac{2TP_{c}}{2TP_{c}+FP_{c}+FN_{c}}$$

(12) $$Jaccard=\frac{TN_{c}+TP_{c}}{TN_{c}+TP_{c}+FP_{c}+FN_{c}}$$

where c denotes a category region. $TP_{c}$ and $TN_{c}$ denote the numbers of the true positive and the true negative pixels in the $c^{th}$ region, while $FP_{c\;}$ and $FN_{c\;}$ are the numbers of the false positive and the false negative pixels in that category, respectively. Two-tailed student *t*-test was used to test the *p*-value between the prediction results of PVSegNet and other methods. And a *p*-value less than 0.05 indicates a significant difference between the predicted results of these two methods. The specific *p*-value results are shown in Table A1, Table A2 and Table A3.

### 3.2. Comparison of Experimental Results

To evaluate the effectiveness of the proposed network architecture, nnUNet, 3D U-Net and CycleGan are used for comparison experiments. The supervised experiments use the annotations of the corresponding portal vein in the H-phase or E-phase images to train the 3D U-Net network. In the other comparison experiments, the portal vein annotation of the P-phase images will be used directly as pseudo labels for training the network on the H-phase or E-phase images. And the same portal vein training dataset was used for all methods. Table 1 shows the performance of different methods in segmenting the portal vein on the H-phase and E-phase images in the test dataset. For fair comparisons, no data enhancement was performed for all experiments in this paper.

In the segmentation results of the portal vein on the H-phase images, the supervised segmentation results of nnUNet can reach 0.832 for DSC and 0.724 for Jaccard, respectively. The supervised segmentation results of 3D U-Net can reach 0.724 for DSC and 0.581 for Jaccard, respectively. Using images *I*^A^ or *I*^V^ and annotations *True*^V^ in the P-phase, 3D U-Net can reach 0.590 and 0.117 for DSC and 0.581 and 0.079 for Jaccard, respectively. The results show that the model trained with only the P-phase image *I*^V^ and *True*^V^ has poor generalization results in the H-phase and can barely recognize the features of the portal vein. The results of the model trained with image *I*^A^ as well as *True*^V^ show that useful information can be learned from H-phase images and ground truths, but the lack of contrast has an impact on H-phase image segmentation. According to the segmentation results of CycleGan, the effect of using only style transfer can reach 0.633 for DSC and 0.482 for Jaccard, respectively. Finally, the DSC value is 0.689 and Jaccard is 0.546 for the PVSegNet method proposed in this study, which is better than the segmentation results in the comparative experiments that also used portal vein annotations *True*^V^.

In the results of portal vein segmentation on the E-phase images, the supervised segmentation results of nnUNet can reach 0.894 for DSC and 0.816 for Jaccard and the supervised segmentation results of 3D U-Net can reach 0.832 for DSC and 0.723 for Jaccard, respectively. Three-dimensional U-Net can reach 0.650 and 0.801 for DSC and 0.500 and 0.680 for Jaccard using images *I*^A^ or *I*^V^ and *True*^V^ in the P-phase, respectively. The results show that the E-phase image is more similar to the P-phase image than the H-phase image, and the model trained on the P-phase images has higher generalization in the E-phase. The results trained on image *I*^A^ as well as *True*^V^ show that different phase vessel shifts still exist when using P-phase ground truth *True*^V^ as pseudo labels. In addition, the segmentation results using only style transfer of CycleGan can reach 0.643 for DSC and 0.493 for Jaccard, respectively. And DSC value is 0.828 and Jaccard is 0.712 for the PVSegNet method proposed in this study, which is superior to the results in other comparative experiments trained with the ground truths of P-phase images.

### 3.3. Ablation Experimental Results

In this section, ablation experiments were conducted on two parts of the proposed PVSegNet network, analyzing the effect of each module of our method and the pseudo label weights on the experimental results.

(1) Effect of each module on experimental results

Table 2 shows the impact of each module of our method on the experimental results when using the ground truths of the P-phase images to guide the portal vein segmentation on the H-phase or E-phase images. The first row shows the results of removing the style transfer module, discriminator D_B_ and pseudo labels; the second row shows the results of removing the pseudo labels; the third row shows the results of removing the style transfer module, i.e., removing the generator G and discriminator D_A_; and the fourth row shows the results of removing the discriminator D_B_. The experimental results in Table 2 show that the results all decrease after removing these structures. And our proposed PVSegNet network contains each structure and achieves the best segmentation performance.

(2) Effect of pseudo label weights on experimental results

Table 3 shows that when different weights are set to construct pseudo labels, the network can obtain different portal vein segmentation results for H-phase and E-phase images. According to Table 3, when $\lambda_{1}$ is set as the weights of 0.1 or 0.7, the constructed pseudo labels can achieve the best results when segmenting the portal vein on H-phase or E-phase images, respectively.

### 3.4. Visualization Results

**Figure 5:**
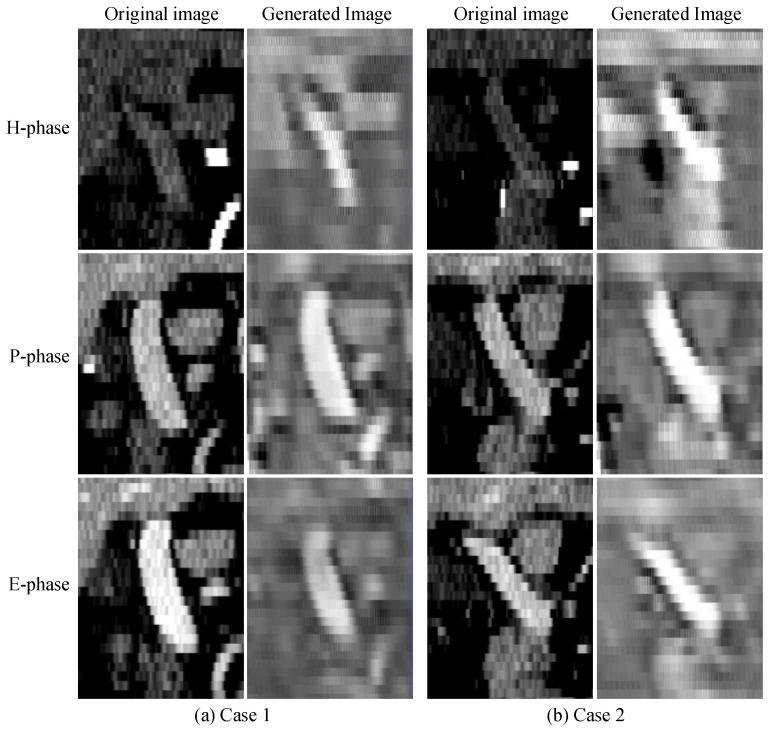
Images generated by the generator G in the style transfer module with similar styles to their respective P-phase image of case 1 (**a**) and case 2 (**b**).

Figure 5 shows the images of two cases from the sagittal plane. Each case was visualized with its original three-phase images and style transferred images generated by the generator G. The transferred P-phase image retains the features of the original portal vein after going through the generator G. There are contrast media on both the E-phase and P-phase images and the portal vein is clear. And after these images are transferred by the generator G, the transferred images also retain most of the information used to segment the portal vein even. The portal vein is not clear in the H-phase image, but after being transferred by generator G, the portal vein is clearer compared to the original image, and the global position information of the portal vein can be extracted despite the loss of some edge information.

**Figure 6:**
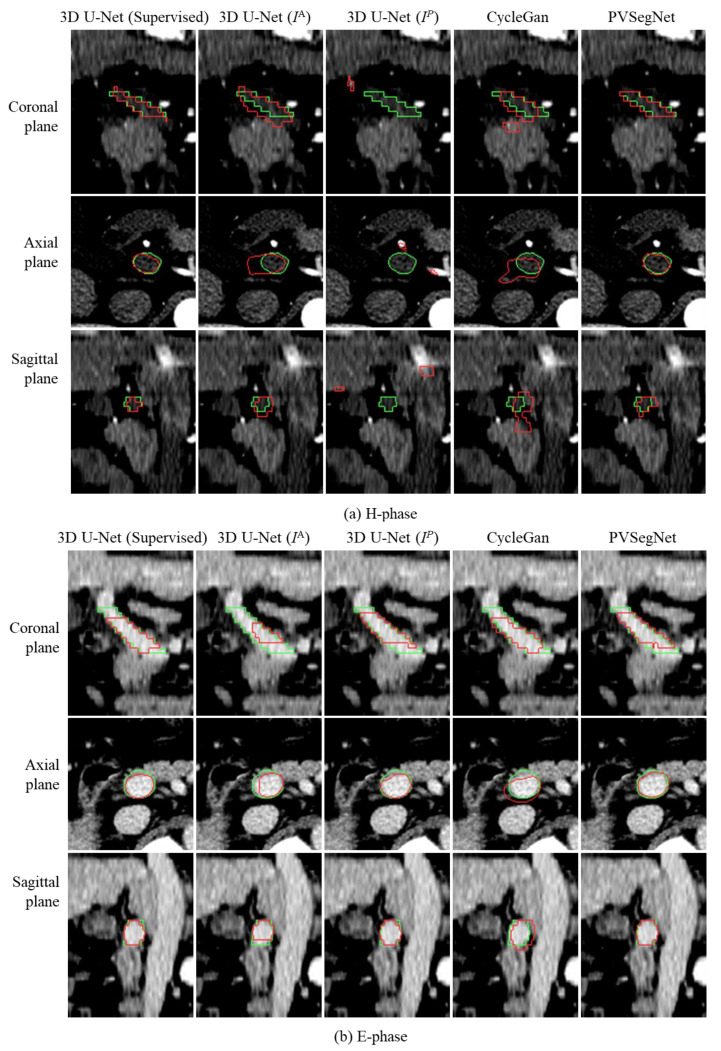
Visualization results of the comparison experiment in the coronal, axial and sagittal planes for the H-phase (**a**) and E-phase (**b**) images, respectively. The green line is the outline of the ground truth and the red line is the outline of segmentation results.

To observe whether the proposed PVSegNet method achieves an effective segmentation of the portal vein, Figure 6 shows the segmentation results on the H-phase and E-phase images, respectively, with the green line indicating the contours of the ground truth and the red line indicating the contours of the segmentation results. From Figure 6, it can be seen that the networks trained directly with the annotations of portal vein images as pseudo-labels, i.e., 3D U-Net and CycleGan, have obviously missed the segmentation phenomenon and cannot segment the complete portal vein, and the segmentation results are slightly worse than those of PVSegNet. In the visualization results, the segmentation results in the E-phase are better relative to those in the H-phase because the vessels in the E-phase are more clearly defined.

## 4. Discussion

Due to the labor-intensive and time-consuming manual annotation of the portal vein on multiphase abdominal CTA images, as well as the challenge of manually annotating the portal vein on H-phase images, we propose a multiphase portal vein segmentation method based on style transfer and pseudo labels. This method can automatically segment the portal vein on H-phase or E-phase images when only P-phase images have portal vein annotations.

The portal vein segmentation network, PVSegNet, mainly consists of a style transfer module and a segmentation module. The style transfer module can convert the H-phase and E-phase images of the same patient to the P-phase images, which makes the segmentation network more easily adaptable to both H-phase and E-phase images. The segmentation module consists of a 3D U-Net network and a discriminator. The discriminator is used to determine whether the segmentation results are similar to the P-phase ground truth, forming a soft constraint on the segmentation network. When using only the annotation of the P-phase images as the pseudo labels to guide the training of the network on the H-phase, as well as the E-phase images, the wrong information is often learned due to the vessel shift. In this paper, we use the annotation of P-phase images and the prediction results obtained from the segmentation network to jointly construct the pseudo labels of the corresponding phase during the training process so as to reduce the effect of vessel shift and obtain more accurate segmentation results. From Table 1, it can be concluded that the proposed method outperforms the model trained directly with the P-phase annotations and H-phase images in the H-phase test dataset. And the segmentation effect in the E-phase images is even better.

However, there is still room for improvement in the study. Firstly, the portal vein segmentation performance in the H-phase images needs further enhancement for the task. The next work will make full use of the annotations of P-phase images to improve the construction of pseudo labels and set strong constraints in the style transfer to achieve the improvement of the algorithm. Secondly, the deep learning segmentation method proposed in this study has been evaluated only on a CT dataset from one hospital, and the future plan is to apply the results of this study to multi-center data for validating the reliability of the algorithm through more clinical applications. Finally, there is a lack of diagnostic studies. Although the portal vein has been segmented, this paper did not use the portal vein to predict portal pressure, and the clinical study will be carried out after collecting more data from relevant patients.

## 5. Conclusions

In this paper, we propose a multiphase segmentation algorithm for portal veins based on style transfer and pseudo labels. Under the condition that only the P-phase portal vein is annotated, this method achieves automatic and effective segmentation of the portal vein in H-phase and E-phase CTA images. The portal vein segmentation network PVSegNet mainly consists of a style transfer module and a segmentation module. The style transfer module converts the E-phase and H-phase images of the same patient to the P-phase images with the same style. The segmentation module consists of pseudo labels guided 3D U-Net network and a discriminator with a soft constraint function. Since the pseudo labels are constructed based on three mutually constrained components, which are annotations on the P-phase image, prediction results obtained from the segmentation network, and soft constraints of the discriminator on the segmentation network, the method can reduce the effect of learned misinformation from vessel shift and distortion. And the method obtains more accurate portal vein segmentation results on both H-phase and E-phase images. This greatly aids clinical practice.

## Figures and Tables

**Table 1:** Performance comparison of different methods in segmenting the portal vein on H-phase and E-phase images in the test set. Inputs with “✔” are used to train the models.

Methods	*I* ^A^	*I* ^V^	*True* ^A^	*True* ^V^	H-Phase		E-Phase	
					DSC	Jaccard	DSC	Jaccard
nnUNet (Supervised)	✔		✔		0.832 ± 0.001	0.724 ± 0.001	0.894 ± 0.000	0.816 ± 0.001
3D U-Net (Supervised)	✔		✔		0.724 ± 0.000	0.581 ± 0.000	0.832 ± 0.000	0.723 ± 0.001
3D U-Net	✔			✔	0.590 ± 0.001	0.437 ± 0.001	0.650 ± 0.001	0.500 ± 0.001
3D U-Net		✔		✔	0.117 ± 0.000	0.079 ± 0.000	0.801 ± 0.001	0.680 ± 0.001
CycleGan	✔	✔		✔	0.633 ± 0.001	0.482 ± 0.001	0.643 ± 0.001	0.493 ± 0.001
**PVSegNet**	✔	✔		✔	**0.689 ± 0.001**	**0.546 ± 0.001**	**0.826 ± 0.001**	**0.712 ± 0.001**

**Table 2:** Effect of each module of PVSegNet on the experimental results. Modules with “✔” are included.

	#	D_B_	G&D_A_	PL	H-Phase		E-Phase	
					DSC	Jaccard	DSC	Jaccard
PVSegNet based	1				0.643 ± 0.000	0.486 ± 0.000	0.758 ± 0.003	0.672 ± 0.007
	2	✔	✔		0.666 ± 0.000	0.485 ± 0.002	0.819 ± 0.000	0.702 ± 0.001
	3	✔		✔	0.648 ± 0.000	0.493 ± 0.000	0.759 ± 0.003	0.624 ± 0.004
	4		✔	✔	0.676 ± 0.002	0.534 ± 0.002	0.823 ± 0.001	0.709 ± 0.002
PVSegNet	5	✔	✔	✔	**0.689 ± 0.001**	**0.546 ± 0.001**	**0.826 ± 0.001**	**0.712 ± 0.001**

Notes: #: Method number; D_B_: Discriminator D_B_; G&D_A_: Generator G and Discriminator D_A_; PL: Pseudo labels.

**Table 3:** Effect of setting different weights for the pseudo labels on the experimental results.

$\lambda_{1}$	H-Phase		E-Phase	
	DSC	Jaccard	DSC	Jaccard
**0.1**	**0.689 ± 0.001**	**0.546 ± 0.001**	0.818 ± 0.000	0.708 ± 0.000
**0.3**	0.665 ± 0.001	0.529 ± 0.001	0.815 ± 0.001	0.698 ± 0.002
**0.5**	0.681 ± 0.002	0.520 ± 0.002	0.819 ± 0.001	0.703 ± 0.001
**0.7**	0.621 ± 0.002	0.474 ± 0.002	**0.826 ± 0.001**	**0.712 ± 0.001**
**0.9**	0.656 ± 0.000	0.498 ± 0.000	0.824 ± 0.001	0.708 ± 0.002

## Data Availability

Not applicable.

## Appendix A

**Table A1:** The *p*-value obtained by comparing the DSC and Jaccard of PVSegNet with other methods using a two-tailed student *t*-test. Inputs with “✔” are used to train the models.

Methods (vs. PVSegNet)	*I* ^A^	*I* ^V^	*True* ^A^	*True* ^V^	H-Phase		E-Phase	
					DSC	Jaccard	DSC	Jaccard
nnUNet (Supervised)	✔		✔		** 4.91 × 10^−22^	** 9.09× 10^−46^	** 1.15 × 10^−26^	2.73 × 10^−1^
3D U-Net (Supervised)	✔		✔		** 4.02 × 10^−3^	** 4.98 × 10^−3^	2.40 × 10^−1^	1.50 × 10^−1^
3D U-Net	✔			✔	** 1.24 × 10^−9^	** 1.38 × 10^−11^	** 1.52 × 10^−33^	** 5.89 × 10^−40^
3D U-Net		✔		✔	** 1.75 × 10^−75^	** 1.36 × 10^−74^	** 1.06 × 10^−5^	** 1.37 × 10^−5^
CycleGan	✔	✔		✔	** 4.15 × 10^−4^	** 3.66 × 10^−5^	** 6.48 × 10^−34^	** 5.15 × 10^−41^

Notes: **: *p*-value < 0.01, significant statistical difference.

**Table A2:** The *p*-value obtained by comparing the DSC and Jaccard of PVSegNet with PVSegNet-based methods using a two-tailed student *t*-test. Modules with “✔” are included.

	#	D_B_	G&D_A_	PL	H-Phase		E-Phase	
					DSC	Jaccard	DSC	Jaccard
PVSegNet Based vs. PVSegNet	1				** 1.59 × 10^−4^	** 4.15 × 10^−6^	** 2.98 × 10^−20^	* 9.82 × 10^−22^
	2	✔	✔		* 1.72 × 10^−2^	** 1.05 × 10^−3^	9.76 × 10^−2^	5.92 × 10^−2^
	3	✔		✔	** 3.47 × 10^−4^	** 2.58 × 10^−6^	** 3.93 × 10^−18^	* 1.61 × 10^−20^
	4		✔	✔	1.06 × 10^−1^	1.10 × 10^−1^	4.31 × 10^−1^	6.63 × 10^−1^

Notes: #: Method number; D_B_: Discriminator D_B_; G&D_A_: Generator G and Discriminator D_A_; PL: Pseudo labels; *: *p*-value < 0.05, statistical difference; **: *p*-value < 0.01, significant statistical difference.

**Table A3:** The *p*-value obtained by comparing the DSC and Jaccard of different $\lambda_{1}\;$ using a two-tailed student *t*-test. For the H-phase, compare the values of $\lambda_{1}$ = 0.1 with other $\lambda_{1}$ values, and for the E-phase, compare the values of $\lambda_{1}$ = 0.7 with other $\lambda_{1}$ values.

$\lambda_{1}$	H-Phase		E-Phase	
	DSC	Jaccard	DSC	Jaccard
0.1			* 4.65 × 10^−2^	* 2.77 × 10^−2^
0.3	** 8.89 × 10^−3^	** 5.24 × 10^−3^	** 6.81 × 10^−3^	** 8.92 × 10^−3^
0.5	** 8.79 × 10^−3^	** 1.58 × 10^−3^	8.66 × 10^−2^	9.18 × 10^−2^
0.7	** 4.60 × 10^−10^	** 3.09 × 10^−11^		
0.9	** 8.12 × 10^−4^	** 1.99 × 10^−4^	5.49 × 10^−1^	4.70 × 10^−1^

Notes: *: *p*-value < 0.05, statistical difference; **: *p*-value < 0.01, significant statistical difference.
